# The cognitive mechanism: safeguarding the future of immunological discovery in the GenAI era

**DOI:** 10.1093/discim/kyag016

**Published:** 2026-08-05

**Authors:** Nigel J Francis, David P Smith

**Affiliations:** School of BioSciences, The University of Melbourne, Melbourne, Victoria, Australia; School of Biosciences, Cardiff University, Cardiff, UK; School of Biosciences and Chemistry, Sheffield Hallam University, Sheffield, UK

## Abstract

Scientific discovery is built on the rigorous interrogation of the unknown. In immunology, exploring novel mechanisms requires researchers to identify what is unknown, reconcile contradictory data through original thinking, and integrate disparate ideas to generate testable hypotheses. Undertaking this process is how students construct their own knowledge and experiences. However, the rapid integration of Generative AI (GenAI) into higher education risks short-circuiting this cognitive process. Time-poor students, facing mounting pressure, increasingly use GenAI to offload thinking onto algorithms, sidestepping the very struggle that develops scientific curiosity and critical judgment. This opinion piece argues that without adequate guardrails, we risk training scientists who simulate competence but lack the capacity for genuine discovery. Taught programmes must be designed with academic integrity in mind and embrace assessment innovations. Rather than engaging in a futile arms race to detect outputs, educators must shift assessment from traditional final products, such as essays and lab reports, to validating the research process itself and assessing directly the capacities that future graduates will need. By assessing research trails, interpretation of raw data, and the thinking process behind GenAI use (identifying gaps, bias, and hallucinations) through viva-style interactions, we ensure that student cognitive effort remains the driver of the work. Ultimately, the quality of future immunological research depends on the integrity of our current assessment methods.

## Introduction

Immunology is often introduced to students as a foreign language full of jargon, CD markers, interleukins, and signalling cascades. Yet, at its core, immunology is a discipline of synthesis that requires navigating ambiguity and contradictory data to formulate original hypotheses. Like any robust scientific endeavour, it demands a high degree of cognitive engagement, something that, if used incorrectly, Generative AI (GenAI) has the capacity to bypass. It must be acknowledged that cognitive offloading is not a fundamentally new challenge; long before GenAI, students could have others complete their coursework, thereby skipping the thinking part. GenAI simply makes this process vastly more efficient, cheaper, and almost universally accessible. Should we fail to adapt our pedagogical frameworks, we risk producing a generation of researchers who simulate competence without genuine understanding, equipped with polished outputs but lacking the critical thinking required to drive future immunological breakthroughs.

### The cutting edge is a cognitive struggle

Scientific insight and discovery come from the friction of struggling with the unknown. In the context of learning, this cognitive load is not a problem to solve; it is the very feature that builds critical judgement. If one becomes overly reliant on GenAI tools for education, they risk encountering one of the main issues with these tools: their architecture.

Large Language Models (LLMs) function as probabilistic engines that excel at consensus but struggle with outliers. While a quick use of GenAI with simple prompts can readily and accurately describe well-established foundational concepts, at the edge of research, where novel immunological discoveries are made, GenAI often becomes less reliable, leading to hallucinations and overconfident but factually incorrect answers. Moreover, GenAI currently lacks the inherent imagination required to synthesize truly new ideas; it is fundamentally limited to the data it was trained on [1]. Genuine immunological discovery relies entirely on investigating the unlikely: a rare cell subset, an unexpected transcriptomic shift, or an atypical immune deficiency. By offloading thinking to GenAI, one bypasses the neural pathways responsible for deep learning, creating a dangerous phenomenon of simulated competence in which high-level essays on complex topics are produced while the individual lacks the ability to explain the mechanistic underpinnings [2].

### Humanistic learning and the value of educators

As GenAI tools inevitably improve, possessing the ability to rapidly access and process vast amounts of information, one might question what educators offer beyond current algorithms. It is true that educators, much like GenAI, can also exhibit an edge effect at the boundaries of their knowledge and may occasionally show overconfidence, even bluffing their way. However, students continue to deeply value human lecturers over GenAI because of the humanistic elements of learning [3]. Educators bring lived experience, contextual understanding, empathy, and scientific intuition that algorithms simply lack. A lecturer does not merely deliver facts; they guide students through the frustration of failed assays, model deep scientific scepticism, and share the realities of laboratory work that cannot be scraped from a database. This human connection, paired with the shared experience of cognitive struggle, is an irreplaceable component of developing independent, resilient researchers.

### Constructive integration of AI in research

Despite its limitations in generating novel concepts, we must acknowledge the vital and constructive role AI will play in future immunology discovery. Tools like AlphaFold are revolutionizing 3D protein structure discovery, identifying epitopes, and predicting antigen–antibody interactions [4]. Machine learning models are already essential for analysing the vast datasets generated by single-cell RNA sequencing, helping to screen thousands of potential antigens to predict which will trigger a robust immune response [5]. Furthermore, integrating high-throughput multi-omics data with computational modelling is helping researchers map the immune system holistically [6].

The development of tools like the AI Co-Scientist promises to help scientists rapidly brainstorm and design experiments. Co-Scientist might be a helpful tool for brainstorming, but it still relies heavily on the user's fundamental knowledge and understanding to initially write a robust prompt. Furthermore, the human scientist must subsequently critically evaluate the AI's outputs for feasibility and suitability. While an AI might suggest an elegant, multistage *in vivo* experiment to investigate an immune pathway, it remains to be determined whether these tools can accurately evaluate the practicality of such a proposal against the stark realities of a specific laboratory's budget and equipment availability. Therefore, our goal should not be to shelter students from technology, but to teach them to engage effectively with these tools while explicitly appreciating their limitations [7].

### Designing assessments: in-person exams vs. authentic process

To safeguard the cognitive development of our students, we must make an intentional shift from valuing the final manuscript, which is highly vulnerable to GenAI, to evaluating and validating the process trail and the act of creation [8, 9]. One simple solution frequently discussed in response to GenAI is a return to in-person exams and invigilated exam halls. We must give a nod to in-person exams: they do have some value, as assessing students in a controlled environment is effectively AI-proof and guarantees that students have acquired a baseline of knowledge without outside interference [10]. We also advocate that fundamental knowledge is critical to use GenAI effectively; you must write a good prompt.

However, in-person exams are ultimately a flawed assessment method. Not only do they put unnecessary stress on students, causing some to perform poorly due to anxiety, but more importantly, closed-book, handwritten exams are simply not representative of the modern scientific workplace. A practising immunologist always has access to literature, raw data, peers, and now, GenAI tools. Therefore, rather than retreating entirely to the exam hall, educators should shift assessment towards authentic tasks, where GenAI is treated as fallible and biased rather than an oracle to be trusted. By auditing or validating the research trail through documents and conversations, we witness the evolution of an idea, the refinement of prompts, and the interpretation of raw data, ensuring that human cognition initiates and drives the work.

### Embracing the ‘fallible’

If we shift the educational relationship to treat GenAI as fallible, this forces the student to think critically, review, critique, validate, and correct outputs. To enforce this dynamic, we require a justification of GenAI usage, where students submit a statement detailing exactly how the tool was used and why it did not compromise the scientific integrity of the work. This allows academics to check GenAI interactions in the same way that a well-documented method enables other scientists to replicate an experiment. This culminates in a professional conversation, or a viva-style interaction, in which students demonstrate their understanding. If the student blindly relied on GenAI hallucinations arising from edge effects or failed to engage with the outputs, they would be unable to defend their specific conclusions. This approach to assessment reinforces the need for verification and ensures the individual is central to the process [11, 12]. It must be acknowledged that these assessment types also have the potential to induce anxiety in students [13]; however, they much more closely reflect the ways professional scientists engage with peers in the workplace, discussing, defending, and developing ideas, and it is a skill worthy of development. Trust can be built between students and academics by providing informal opportunities to discuss and develop ideas during tutorials, seminars, and by establishing mechanisms to set expectations and address misconceptions. Further opportunities to practice viva-style assessments, followed by effective feedback, are also key to helping students understand that they are engaging in a dynamic conversation with an academic who is keen to explore their knowledge [14]. Trust building helps students realize that these assessments are not witch hunts in which staff are trying to catch them out, but rather a supportive mechanism for them to gain an appreciation of the assessment criteria and expectations, thereby enhancing assessment transparency [15]. Additional advantages of conversational-style examinations are that students can have access to notes, just as they can bring their thesis into a PhD viva, and misconceptions can be quickly identified and discussed, helping a student who may have misinterpreted a question to correct themselves during the assessment.

The pedagogical strategies we deploy today have profound, long-term implications for the field of immunology. The habits formed during rigorous assessments, strict verification, deep scientific scepticism, and a relentless focus on interpreting raw data feed directly into the vital protocols of professional research integrity. The cognitive discipline required to manually dissect a flawed hypothesis is the exact same discipline required to troubleshoot a failing assay or identify a genuine breakthrough amid a sea of noisy data. A student who is permitted to passively accept a GenAI-generated hallucination today is deprived of the training needed to become a rigorous, independent scientist tomorrow.

## Conclusion

As we navigate this rapid technological shift, GenAI stands as a powerful enabler, allowing students to focus on higher-level learning, provided they possess the foundational knowledge required to evaluate the outputs. However, we must remain vigilant to ensure it never replaces the fundamental human curiosity that drives genuine scientific discovery. The future of immunology relies on researchers who are not merely operators of algorithms, but profoundly deep thinkers capable of interrogating the unknown. By explicitly teaching students to use tools like AI Co-Scientist responsibly and intentionally preserving the cognitive struggle inherent in scientific training, we equip future immunologists to confidently embrace new technologies. In doing so, we ensure that the human scientist's critical, synthesis-driven mind remains central to immunological progress.

## Data Availability

No new data were generated or analysed in support of this research.
